# Health workers perceptions and attitude about Ghana’s preparedness towards preventing, containing, and managing Ebola Virus Disease

**DOI:** 10.1186/s12913-017-2225-0

**Published:** 2017-04-12

**Authors:** Philip Baba Adongo, Philip Teg-Nefaah Tabong, Emmanuel Asampong, Joana Ansong, Magda Robalo, Richard M. Adanu

**Affiliations:** 1 Department of Social and Behavioural Sciences, School of Public Health, University of Ghana, Box LG 13, Legon, Accra, Ghana; 2 World Health Organization Country Office for Ghana, P.O. Box M.B.142, Accra, Ghana; 3 Department of Population, Family and Reproductive Health, School of Public Health, University of Ghana, Box LG 13, Legon, Accra, Ghana

**Keywords:** Ebola Virus Disease, Perception, Preparedness, Health Workers, Health System, Ghana

## Abstract

**Background:**

Ebola virus is highly infectious and the disease can be very fatal. The World Health Organization has declared the 2014–2015 Ebola Virus Disease outbreak a Public Health Emergency of International Concern. In response to this, preparations were made in various health facilities and entry points across Ghana. This study explored health workers perceptions, and attitude about Ghana’s preparedness towards preventing and containing Ebola Virus Disease.

**Methods:**

We conducted a qualitative study in five (5) of the ten (10) regions in Ghana. Five focus group discussions (*N* = 44) were conducted among nurses; one in each region. In addition, ten (10) health workers (2 in each region) who are members of regional Ebola Virus Disease task force were recruited and interviewed. In the Greater Accra, Volta and Western regions that have ports, six (6) port health officials: two in each of these regions were also interviewed. The interviews were recorded digitally and transcribed verbatim. Thematic content analysis was used to analyze the transcripts with the aid of NVivo 10 software.

**Results:**

The results of this study showed that Ghanaian health workers perceived the screening at various ports as important and ongoing but felt that the screenings at in-land ports were being undermined by the use of unapproved routes. Training of health workers was also being carried out in all the regions, however, there was a general perception among 33 out of 44 nurses that majority of health workers have not received training on Ebola Virus Disease prevention and management. Logistical challenges were also reported as some health facilities did not have adequate Personal Protective Equipment. In facilities where equipment was available, they were stored in places which are not easily accessible to health workers at all times of the day. Human resource preparation was also perceived to be a challenge as health workers (38/44 of nurses) generally expressed fear and unwillingness to work in Ebola treatment centres in the event of an outbreak in Ghana.

**Conclusions:**

Our study concludes that preparatory work for Ebola Virus Disease prevention and containment in Ghana is perceived as inadequate by health workers. Ghana needs to strengthen preparation in the area of training of health workers, provision and accessibility of Personal Protective Equipment and incentives for health workers to better position her to contain and manage any Ebola Virus Disease outbreak.

## Background

Ebola Virus Disease (EVD) outbreaks are not new in Sub-Saharan Africa [1]. EVD was first recognized in the year 1976 when two epidemics occurred almost simultaneously in Zaire (Democratic Republic of Congo) near River Ebola, and Sudan. Since then, more than 20 outbreaks have occurred, mostly in Equatorial Africa region [1]. On 22 March 2014, the Guinea Ministry of Health notified the World Health Organization (WHO) about a rapidly evolving outbreak of EVD [2]. Retrospective epidemiological investigations revealed that the first case of the disease probably occurred as early as December 2013 when a two-year-old girl from Guéckédou prefecture in the forested region of south-eastern Guinea died from symptoms suggestive of EVD [2].

This 2014–2015 outbreak has been declared a Public Health Emergency of International Concern by the World Health Organization in August 2014 [3]. The disease that started in rural Guinea spread to countries such as Liberia, Mali, Sierra Leone, Nigeria, United States of America and Spain. A total of 28, 646 confirmed, probable, and suspected cases of EVD have been reported in all the affected countries since the outbreak began with 11,323 deaths [4]. EVD affects people of all ages but the heaviest toll has been reported to be on the most active segment of the population (15–44 years), [5]. The rapid way the disease is spreading calls for measures to be taken by unaffected countries in readiness for any outbreak. This is even critical for countries within the West African sub-region because of high trans-border activities with neighbouring countries with the outbreak [6].

In response to the 2014–2015 EVD outbreaks, Ghana has established an inter-ministerial committee under the leadership of the Minister of Health and national preparedness and a response plan was developed in August 2014. The plan is structured around five thematic areas: planning and coordination; surveillance; situation monitoring and assessment; case management; social mobilization and risk communication; and logistics, security and financial resources [7]. Ghana Health Service, and the Ministry of Health have also intensified social mobilization and risk communication on EVD across the country. In addition, EVD response teams have been established at national, regional and district levels. To equip stakeholders with knowledge on how to manage an outbreak, training of frontline health workers was also carried out across Ghana. Furthermore, national and regional EVD treatment centres were established [7] and Personnel Protection Equipment (PPE) supplied to health facilities. As a measure to prevent the importing of EVD and to promote early detection of EVD among international travellers, screening exercises have also been undertaken at various entry and exit points in Ghana.

During an EVD outbreak, health workers who take care of infected people are at higher risk of infection [8]. Records available show that during this current (2014–2015) EVD outbreak health workers are between 21 and 32 times more likely to be infected with Ebola than the general population. The disease has infected about 881 health workers and more than half of them have died [9]. In the 2000-2001 EVD outbreak in Uganda, 224 individuals were reported to be infected with the disease, however 14 of those who died were nurses [10]. Lessons from previous EVD outbreaks have showed that three core interventions have contributed to containing those outbreaks mostly in equatorial Africa: exhaustive case and contact finding, effective response to patients and the community, and preventive interventions [11]. Perception about a country’s preparation has been found to have close association with perceived risk of infection among health personnel which also determine their willingness to take care of patients. Historically, perception about the preparation of the health system to contain an outbreak and perceived higher risk of infection among health workers and their families have resulted in their hesitation and refusal to provide care to patients during the outbreak of SARS [12–14], the early years of HIV [15–17] and other catastrophic disasters [18–20]. Unwillingness to take care of patients may have serious implications for the response capacity of a country and may undermine containment efforts. Hence, good preparation and having trained health personnel who are willing to take care of EVD patients is essential in an outbreak.

Although Ghana has not had a confirmed case, the high trans-border activities between Ghana and neighbouring West African countries put Ghana at risk of an outbreak [6]. Adequate preparation is required to manage cases and contain any outbreak. In Liberia, Sierra Leone and Guinea, it has been reported that one factor that made containment of the outbreak difficult was that the health system in those countries was not well-prepared to manage and contain an outbreak [21, 22]. Since Ghana has made some preparations towards managing and containing a possible outbreak [7], it was important to explore the views of health workers who are key players in this preparation. This study therefore explored health workers perceptions and attitude about Ghana’s preparedness towards preventing, controlling and managing EVD.

## Methods

### Study design

We employed descriptive qualitative study design in this research. Since no previous studies have been undertaken in Ghana on EVD, a qualitative research approach was deemed appropriate to gain deeper insight on health workers perceptions about Ghana’s preparedness towards preventing and containing EVD. We used phenomenology strategy in qualitative enquiry. Phenomenology approach to qualitative research allows participants’ to share their perceptions, feelings, and lived experiences in the community and how these experiences affect their perspectives about a given situation [23].

### Study area

The study was conducted in the Republic of Ghana which is located in West Africa's Gulf of Guinea only a few degrees north of the Equator. Ghana shares borders with the three French-speaking nations of Côte d'Ivoire to the west, Togo to the east, and Burkina Faso to the north. To the south of Ghana are the Gulf of Guinea and the Atlantic Ocean. Ghana has a population of 24,658,823 with an annual growth rate of 2.4% [24]. The nation has ten administrative regions which are further divided into metropolitan, municipality, districts and sub-districts. There are about 75 ethnic languages spoken in Ghana, however, English is the national medium of communication. Majority of Ghanaians are Christians (over 70%), Islam (a little over 17.5%) and the remaining adhere to traditional religion and other faiths [25].

In Ghana, health service delivery follows a three-tier arrangement: primary, secondary and tertiary levels. The primary and secondary level cover health facilities at the sub-district, district and regional levels. The tertiary level health care is mainly provided by the four teaching hospitals located in Northern, Ashanti, Central and Greater Accra regions of Ghana. In 1996, the Ghana Health Service and Teaching Hospitals Act (ACT 525) was passed to separate health policy formulation from operational management of health service delivery. This Act mandates the Ministry of Health to formulate health policies whilst the Ghana Health Service has the obligation to implement these health policies. An estimated 52, 258 people are formally working in the health sector in Ghana, of which 81.5% are employed in the public sector. Ghana’s doctor-patient ratio is approximately one doctor to 15,259 population [26]. However, nurse to population ratio is reported to stand at one nurse to 1251 population [27]. Even though the doctor-patient and nurse-patient ratios have progressively improved over the years, rural–urban inequities still exist, as majority of the health workers are reported to be working in urban areas [28]. There are also about 21, 791 registered traditional medical practitioners and 367 registered traditional birth attendants practicing in Ghana [26].

This study was however conducted in five of the ten regions in the Republic of Ghana with a combined population of 15,764,171. The five regions included in the study are the Greater Accra, Western, Volta, Ashanti and Northern. The Greater Accra region (GAR) and Ashanti region (AR) are the two most densely populated regions in Ghana with populations of 4,010,054 and 4,780,380 respectively, and corresponding population densities of 1236 and 196 per square km (GSS, 2012). The Volta Region (VR) has a population of 2,118,255 whilst the Western Region (WR) has a population of 2,376,021. Northern Region (NR) has a population of 2,479,461 and is the largest among the three regions located in the northern part of Ghana [24].

The selection of these regions was based on rural-urban dynamics, population density and entry and exit points. The Greater Accra and Ashanti regions were selected based on their high population densities. High population density has been noted to increase both animal-to-human and human-to-human EVD transmission [29]. Also, the Greater Accra region is the main sea and air entry point in Ghana. The Volta Region shares a border with Togo at Aflao whilst the Western Region shares boundary with Cote D’ Ivoire at Elubo and also serves as major sea port. The two Regions (Volta and Western) were therefore selected because they serve as entry and exit points in Ghana. The Northern Region also shares a border with Cote D’Ivoire to the west of Bole District.

### Selection of participants and data collection

We included males and females who were adults 18 years and above as required by law for informed consent [30]. Purposive sampling technique was used to select the nurses, EVD task force members and the port health officials. Some data collection strategies used in phenomenological-based research include; interviews, conversation, observations, group discussion and analysis of personal text [31, 32]. In this study, we utilized two of these data collection strategies: focus group discussions (FGDs) and in-depth interviews (IDIs). We conducted five (5) FGDs among nurses; one in each of the five regions. Each FGD was made up of between 8 and 10 nurses with a total number of participants as 44 nurses. Each discussion was facilitated by a moderator with a note-taker recording the discussion. During FGDs, each participant was given the opportunity to contribute to any question asked before moving to another question. Generally, it took between 60 and 90 min to complete a FGD. We also interviewed ten (10) health workers who were mostly members of regional Ebola taskforce. These health workers were Deputy Directors of Health Services in-charge of public health for each region (Public Health Physicians: 5), Regional Disease Control Officers (3), Biomedical Scientist in-charge of Regional Hospitals (2). In addition, we interviewed six port health staff: two each in the Greater Accra, Western and Volta regions. The port health officials comprised of two medical officers and four nurses. Semi-structured IDIs and FGDs guides designed in English were used in the data collection. The guides explored areas such as perception of Ghana’s preparedness covering screening at various points of entry, training of health workers, provision of PPE, availability of treatment centres, attitude towards suspected cases, and willingness to accept posting to EVD treatment centres (see appendix 1 for the guides used for the study). Graduate research assistants were recruited, trained and deplored to the various regions for the data collection. The data collection period was February, 10-March, 3 2015. The data collection was done simultaneously in all the five regions.

### Data analysis

Large qualitative data set is often generated when a researcher employs phenomenology approach to research. This data set come in the form of interview notes, tape recordings, field notes and jottings which are often analysed together [33]. In that light, all FGDs and IDIs were digitally recorded with the participants’ permission and transcribed verbatim. Field notes were also transformed into data documents within a day of the IDIs or FGDs. These notes, as well as transcriptions, were anonymised, and no identifying information were included in the notes. Each participant was assigned a unique identification number. All transcripts were reviewed by an independent person. In the review, the independent person listened to the various recorded voices and compared the voices with the transcripts. Qualitative narrative data in English were then entered into a word processor (Microsoft Word) and imported in a format that allows coding of the interview transcripts in NVivo 10.

Thematic analysis was employed in analysing the data. Thematic data analysis process consists of three interrelated stages namely data reduction, data display and data conclusion-drawing/verifying [34]. Guest, Macqueen & Namey, also summarizes the process of thematic analysis as consisting of reading through textual data, identifying themes in the data, coding those themes, and then interpreting the structure and content of the themes [35]. Using this method, a codebook was first developed, discussed and accepted by the researchers. Nodes were then created within NVivo using the codebook. Line-by-line coding of the various transcripts was done as either free or tree nodes. Double coding of each transcript was carried out by two of the authors. Coding comparison query was used to compare the coding and a kappa score (measure of inter-coder reliability) was generated to compare the coding that was done by the two researchers. These scores were found to be between 0.8 and 1.0, indicating a higher level of agreement. Matrix coding strategy (query in NVivo) was performed to compare the coding against nodes and attributes. This made it possible for us to compare and contrast within-group and between-group responses. We also used the explore function in NVivo to draw a framework capturing various aspects of the data set and their attributes and nodes.

## Results

Using phenomenological approach to qualitative enquiry require the researcher to provide a summary of the findings in the form of themes. These themes should emerge from the data and should reflect the key issues from participants. In doing that, the researcher is expected to remain faithful to the participants by employing what is known as bracketing in phenomenology. Bracketing is used to minimize personal biases in presenting the findings of a study [36, 37]. This is often necessary because of the close relationship between the researcher and the researched during the research process. Hence, we summarised the results of our study by the themes that emerged from the data and supported the narratives with illustrative quotes from respondents as required in phenomenology [31, 36]. Bracketing was also used to ensure a fair presentation of the results.

### Perceptions about Ebola Virus Disease screenings at various ports

One of the strategies for containing EVD is early detection and isolation of suspected cases through screening of international travellers. Interviews with port health staff revealed that screening of travellers was being conducted at the various entry points (air, sea, and land) in Ghana. The study showed that in addition to taking their temperatures using a non-contact (laser) thermometer, travellers were made to fill a health declaration form. This form requires all travellers into the country to provide information on their travel history, their contact address whilst in Ghana and to disclose their state of health regarding any of the non-specific symptoms of EVD. Respondents illustrated this below:

“*So as the person is coming out we take their temperature with the non-contact thermometer. We take their temperature one after the other. We also give them a form called health declaration form to fill. This form enable us to know the travel history of the passengers. The form will also collect information on your nationality, age, your address, where you are going, and your contact number. We also ask you to declare your health status regarding the signs and symptoms of Ebola*” (IDI, Port Health Official, WR).

*“For all people coming to Ghana, we check their temperature with a laser thermometer. We also take your history such as fever, coughing, headache, vomiting, sore throat, body weakness, pain, diarrhea and bleeding from any part of the body. If anybody should have any of the signs indicated, we quickly isolate the person to a place and then we conduct our investigations properly. If the person is coming from an Epi-zone, I mean where there is an outbreak of the disease, you are detained and the EVD response team informed*” (Port Health official, GAR).

Nonetheless, port health officials at in-land ports reported that screenings were being undermined by the use of unapproved routes at border towns. This is because Ghanaians who live at such border towns frequently visit relatives across the neighbouring countries and enter the country through unapproved routes. This was particularly reported in the Volta region at Aflao (border town to Togo) and in the Western region at Elubo (border town to Cote d’ Ivoire)

*“… here [Elubo] they have relatives across Cote d’Ivoire and they normally go here and come back without our knowledge because they use the other access routes that do not have port health officials*” (IDI, Port Health official, WR).

*“You know how the settlement pattern is here, some people live in Aflao (Ghana) and have their relatives across the border in Togo, so it is difficult to control the trans-border movements. They know the terrain very well and will cross and come back without people knowing*” (IDI, Port Health Official, VR).

### Perceptions about training of health workers

The study solicited respondents’ perceptions about the level of Ghana's preparedness to deal with a possible EVD outbreak regarding the training of frontline health workers. There were mixed perceptions regarding the level of training that has been organized across the country. Some respondents were of the view that majority of health workers have been trained across Ghana. This view was mostly held by members of the Ebola taskforce across the regions as illustrated by respondents:

*“You know training has been done for all facilities and within the facilities they also have the response team and they are supposed to set up holding rooms, though I will say not all of them have that [holding rooms]”* (IDI, EVD Response Team Member, GAR).

*“The Ebola response team members in this region have organized training for various people in our borders, health workers, immigration and port health staff”* (IDI, EVD Response Team Member, VR).

*“Yes, our medical superintendent and the matron [Head of Nursing] attended the training and it was such that they were then supposed to come back and train us here at the hospital. So when they came back they organized training for us, which lasted for three days. They educated us on so many things about the Ebola, the definition of Ebola, what you can do to prevent Ebola” (*IDI, Health Worker, NR).

*“….And I also think that we have gone far because if we have been able to produce some level of health workers who are prepared to go outside and support in managing the outbreak. I think we have done quite a lot and I know that at least from this region, about four people were part of the team that went out on the ticket of ECOMOG to Sierra Leone and Liberia* to work” (IDI, EVD task force member, AR).

This notwithstanding, some health workers mostly nurses (33 out of 44 nurses in FGDs) were of the view that majority of their colleagues have not received any training on Ebola. Respondents observed that only a few people have been trained on Ebola preparedness, management and prevention. Therefore in their view the preparation regarding training of health workers was inadequate especially training for those at the lower level of the workforce as observed below:

*“As for my ward, nobody was selected for any training on Ebola. I am in the surgical ward. I only heard that people were selected for training, whether they actually selected the people or not I do not know but nobody was selected from my ward”* (FGD, Nurse, NR).

*“For my facility I don’t think they [Ghana] is well prepared; many of us health workers are not trained, no isolation centres in our health facilities and yet we keep hearing that we are prepared”* (FGD, Female, Nurse, GAR).

*“We are not secured, looking at the situation now…majority of us nurses have not been trained. If nurses have not been trained then what about the cleaners who have not been trained, what about orderlies?”* (FGD, Female, Nurse, WR).

*“Here is the case we don’t even know those who were trained and what of if the person is off [duty] or is on sick off, so who will be there to attend to that patient? We are health professionals so the best thing is that those who went for the training must come and train those who could not go in their facility so that we all will be abreast with how to manage Ebola, not that one person will know so that if that person is not there, then there will be nobody to attend to the patient*” (FGD, Female, Nurse, GAR).

There was a general belief among respondents that only some clinical staff have been singled out and given training on Ebola. These selected workers get the opportunity to attend all Ebola workshops to the detriment of other supporting staff. Respondents were of the view that all people working in any hospital and other places where there is a possibility of coming into contact with Ebola cases should be trained. They therefore suggested that training on Ebola should be expanded to include health professional that provide preventive services such as Public Health and Community Health Nurses as illustrated:

*“….I think they should educate everybody especially those health workers in the community like Community Health Nurse or Public Health Nurse; they do a lot of domiciliary service to the community. They should be involve in the workshop*” (FGD, Nurse, WR).

*“It is important for everybody working in the hospital to be trained on Ebola including cleaners because good disinfection of the floor is also required in preventing the spread of Ebola. Now that even nurse are not trained, I doubt if the other health workers have been trained”* (FGD, Nurse, VR).

### Provision of personal protective equipment and treatment centres

The study further explored health workers perception concerning the provision of personal protective equipment (PPE) for managing Ebola cases. The results of the study showed that respondents were of the view that PPE supply to various health facilities was inadequate. This view was unanimous across all regions in FGDs and majority, 8 out of 10 (80%) of taskforce members also had similar views on the supply of PPE. The following are illustrative quotes from the respondents to buttress their claim:

“*The hospital has brought some five Ebola protective gowns to the hospital and at every corner they have pasted information about Ebola in my facility that’s what has been done so far…the gown are just five in number*” (FGD, Nurse, AR).

*“… we do not have the Ebola ones but we have our normal nursing ones like the gloves, small cap, our aprons and mask but as to the Ebola gowns, boots and masks we do not have them”* (FGD, Nurse, NR).

The results of the study further revealed that even in facilities that have been provided with PPE, these equipment are not easily accessibly according to respondents. These PPE are often kept in cupboards and locked. The keys to such cupboards are often in the possession of the unit in-charges. However, these unit “in-charges” are not available at the unit all time of the day. Respondents also observed that the lack of PPEs may either put health workers at risk of getting infected or may cause a delay in attending to patients suspected to have EVD as illustrated:

*“…even like our sister said the PPEs are in someone’s office locked, if even the person has closed and a case comes in the evening how do we handle it?”* (FGD Nurse, NR).

*“….As for the PPE, these equipment are in the in-charge office and the key is kept by her. So you have to call the in-charge when there is a case to come and release PPE before you can attend to the patient”* (FGD, Nurse, AR).

*“Waoow! I will feel very unsafe especially if I am supposed to care for that patient knowing very well that the gadgets and things needed to care for that client are not available. I will feel very unsafe and may even contemplate resigning but we were told that working at the Ebola treatment centre will be voluntary*” (FGD, Nurse, NR).

*“If the equipment for protection are not available, then I will be afraid to attend to the patient. I can get infected without the PPEs, so I will wait till the time that the protective equipment are available before attending to any suspected case*” (FGD, Nurse, WR).

Another area explored in this study is the perception about the availability of treatment centres for Ebola cases in the various regions. Participants emphasized that this area requires attention, as many health facilities did not have holding places for suspected Ebola cases. Even where places have been designated, the rooms are not well furnished to make them proper holding centres for Ebola patients. This was well-entrenched and undisputed by all participants across all regions as illustrated:

*“….I have seen that at least one treatment centre is complete. That one I know it is there and attempts are being made at all regional levels to get treatment centres. We have even directed that every district hospital should at least identify a holding area and prepare it. Even though they identified the centres, the places are really not prepared, I think a lot has to be done”* (EVD task force member, VR).

“*We do not have the treatment centre. I say no because as we speak now even in this municipality, there is no single holding room not even to talk about getting people to work there. Even at the ports we do not have holding rooms to even get people to work. At the regional level we do have such a centre but not in the municipalities*” (IDI, Health Worker, AR).

### Attitude of health workers to Ebola Virus Disease prevention, case management and containment

The study also revealed that there has been an increased in hand hygiene practices among health care providers following their sensitization on the disease. However, the use of common instruments such as thermometers, sphygmomanometers and stethoscopes on patients was of great concern to nurses in this study.

*“This time we have been provided with hand sanitizers and people now take hand washing very seriously [sic]” (*FGD, Nurse, VR).

*“Since you can get it through body fluid, sweat for example, so you remove the hand band (cuff) of the sphygmomanometer from somebody’s hand and then another person comes and also sits and then you do the same thing, I think that can facilitate transmission”* (FGD, Female, Nurse, GAR).

*“Even without this Ebola thing, it was actually pathetic to see that one cuff can be used for several patients for a month or two without even washing it”* (FGD, Nurse, WR).

One area of discontent among health care workers in this study was the advice to health workers to stay at home should they experience any of the non-specific signs or symptoms of Ebola and monitor themselves and report to the appropriate authority when their condition is not getting better. This instruction appears to have been perceived to mean that health workers may be neglected should they contract the condition whilst caring for patients with Ebola as illustrated by a nurse:

*“As part of the control measures to curb the virus, we were told that if a health worker, by chance comes into contact with a suspected case and you start running temperature (have fever), you should stay at home as a health worker and monitor yourself and when the temperature starts rising, you tell the regional team. I don’t think you are being fair to us at all because even with the other patients, we go to monitor them. So in the same vein, I think if a health worker per chance comes into contact with a suspected EVD patient, they should monitor the health worker and do as we do for any other person but not to say the health worker should stay at home with her family and monitor herself. I think this should be revisited”* (FGD Nurse, GAR).

The results of the study also revealed that health workers saw Ebola as a deadly disease and as such will not accept postings to Ebola treatment centres in the event of an outbreak. Majority (86.4%) of nurses in this study expressed their unwillingness to work in Ebola treatment centres. This unwillingness to attend to Ebola cases was also believed to be common across all cadre of health personnel whose work may require direct contact with patients or their body fluids such as doctors and laboratory workers. Respondents recounted two classical occasions when suspected Ebola cases were taken to the hospital and blood samples had to be taken for laboratory investigation to illustrate the unwillingness of other cadre of health workers to take care of Ebola cases. In those classical situations, all the laboratory workers deserted their post as illustrated by Ebola response team members:

“… *you know everybody fears Ebola, so if you ask any health worker if they are willing to treat Ebola now, they say no and they are all running away. It is only a few staff who are willing and dedicated…. The last two cases that we sent blood samples of suspected Ebola cases to Noguchi [research centre equipped to conduct confirmatory test for Ebola in Ghana], one was from Komfo Anokye [Teaching hospital in Ashanti region] and it took them six hours to take the blood sample. Who to go and take the blood sample was a problem”* (IDI, EVD Response Team Member, AR).

*“One time there was an incident at the A&E [accident and emergency], a taxi driver brought in a patient and then the nurses triaged the patient alright and then after a while it was like most people have gotten in contact with the person and then at a particular point they noticed it was a suspected Ebola case and then everybody including doctors, nurses and other patients started running here and there”* (FGD, Nurse, AR)

*“I will not accept to work in Ebola treatment centre, because it is a very deadly condition. Even if you are my friend and you agree to work there, for that period we [will] cease to be friends until Ebola is over*” (FGD, Nurse, GAR).

The study revealed that even with the provision of adequate PPE, some health workers are still afraid to work in Ebola treatment centres. This fear emanates from the belief that PPEs do not offer full protection against Ebola because health workers who used PPE in countries with the outbreak were still infected with the disease as illustrated:

*“Sir, in Liberia, Nigeria and Sierra Leone, several health workers who used PPE as you are referring to still got [infected with] the condition and some were not lucky and died, so how sure can you be that we will not get the condition even after using the protective items. It is a big risk and I am too young to take that risk”* (FGD, Female, Nurse, VR).

*“I feel very scared and unsafe even with protection. I am the first born in my family, and I don’t think my family will allow me to volunteer to work at EVD treatment center where I can easily get the condition”* (FGD, Nurse, NR).

The fear of working in Ebola treatment centres was unanimous in all FGDs with nurses in all the regions. A nurses summarize this in the following interview in Greater Accra region of Ghana:

*Interviewer: What is your opinion about accepting to work in an EVD treatment centre?*

*Respondent: God forbid, no no it won’t happen I have to stay alive, God did not say I should love my neighbour more than myself, he says love your neighbour as yourself, so I have to be alive to treat the other thousands of patients around who are with other conditions, ahhh! Please I will run”* (FGD, Nurse, GAR).

Nonetheless, some nurses in FGDs indicated their willingness to accept posting to Ebola treatment centres if incentives are provided. To them the risk of taking care of Ebola patients was too high. Hence, to agree to work in EVD treatment centres will depend on the magnitude of the incentive that is available. Nonetheless, they were in the minority as only 6 out of 44 participants (13.6%) in the FGDs indicated their willingness to work in EVD treatment centres if adequate incentives are provided as illustrated:

*“If our people [Ghana Health Service] were to come out in the first place to really outline the benefits you will get if you accept to work in an Ebola centre. Those who have children. I mean people with dependents, what they will get if they should die in the course of taking care of Ebola patients….So there must be some incentive package or insurance for people who accept to work in Ebola treatment centre”* (FGD, Nurse, GAR).

“*If we are assure[d] of some insurance package for people who accept to work in such areas, then I may accept to do that because it is very risky to take care of Ebola patients because we have read on the internet that several health workers have died from the condition*” (FGD, Nurse, AR).

Respondents suggested a formal documentation of the insurance package for people who agree to work in EVD treatment centres. Any verbal assurance was deemed unacceptable by respondents. Respondents also indicated the need to consider the families of people who will agree to work in EVD treatment centres in determining the incentive package. This is because of the high risk of infecting family members and the possibility of stigmatization of family members in the community. The following quotes illustrate these points:

*“And if I am assured that if you care for the patients, you will be given an insurance so that in case you die you can die peacefully but if it is not on paper but verbal that if you die this is what they will give you, then, I am sorry I cannot work there”* (FGD, Nurse, NR).

*“I will discuss that with my family first and I don’t think they will agree because I can get it and infect them as well. People in the community may even avoid my family members”* (FGD, Health Worker, WR).

## Discussion

In phenomenology approach to qualitative research, the discussion section involves interpretation of the findings of the study, relating it to previous research, personal experience and sometimes commentaries by other researchers. It is also sometimes necessary to develop a tentative theory from interpretation and linkages from the findings [36]. Therefore, the discussion of our study cover the interpretation of the main themes that emerged from the data, comparing the findings with previous studies, drawing the linkages from which a frame work has been developed as a tentative theory on how the various factors interrelate regarding Ebola and the health system.

### Perceptions about screening of international travellers

Generally, the study showed that screening is being carried out at all three ports (sea port, airport, in-land port). Despite this, the high trans-border activities between Ghana and neighbouring countries and the availability of several unapproved entry points along the frontiers poses a threat to the screening efforts in Ghana. The weak control of movement across the frontier, entwined with a weak health system may hinder the ability to detect cases of Ebola being imported into the country. In Ghana, it has been well documented that an infectious disease like cholera was imported into the country through the entry point to Ghana and with a weak health system that infection spread very fast to several communities due to lack of emergency preparedness [6, 38, 39]. The 2014–2015 EVD outbreak though started in rural community has spread to infect people in Spain, United States of America, Nigeria and Senegal. In all these countries, the disease was reported to have been imported from Liberia, Guinea and Sierra Leone [22]. Nigeria’s first case of Ebola, confirmed in July 2014 was the first time the virus had spread by air travel, and this strongly suggests that any city with an international airport is at risk of an imported case [21]. Therefore, continuous screening of international travellers as observed in this study is important for all countries with international airports. Nevertheless, regarding the use of unapproved routes at in-land ports, effective collaboration between port health officials at Ghana side of the border and their counterparts at neighbouring countries will be required. This collaboration will also strengthen cross-border contact tracing and screening of travellers in the wake of an outbreak. Cross-border contact tracing is reported to be more effective than a single country contact tracing when EVD occurs in multiple countries simultaneously [5].

In addition to the entry screening that is being practiced in Ghana, exit screening are often recommended for countries with outbreak [3]. Generally, it has been reported that exit screening are more effective than entry screening [40]. Both entry or exit screening increases vigilance within countries and improves early detection of imported cases of EVD [41]. Taking the travel history of international travellers as well as history on the non-specific signs of EVD such as headache, diarrhoea, and cough are important measures to facilitate early detection of cases as being done in Ghana. This strategy however, relies on individuals to voluntarily provide this information. This strategy may be less effective because of the voluntary nature of it. As observed by Ross, Olveda & Yuesheng, many travellers may refuse to volunteer such information when they are aware that their trip may be cancelled and that they could be quarantined as well if they disclosed signs and symptoms that may be suggestive of EVD [42].

### Perceptions about training of health personnel

The results of the study showed that efforts are being made to train health workers across Ghana. Nevertheless, this was seen to be inadequate as many frontline health workers are yet to receive any training across the regions. By the nature of EVD, every health worker requires basic training particularly on how to handle suspected EVD cases. This is very crucial as it could help prevent cross infection to other patients and the health worker in the health facilities (nosocomial infection). This is because suspected EVD case(s) could report to any facility at times that trained personnel in the various facilities are not present. In previous Ebola Haemorrhagic Fever outbreak in Uganda, nosocomial (hospital-acquired) infections were reported to often occur outside Ebola Haemorrhagic Fever treatment wards because health workers in those areas were not trained on how to care for suspected cases [43]. Drawing lessons from this observation in Uganda’s Ebola Haemorrhagic Fever outbreak, it is important to train every health worker on how to identify and take care of suspected EVD patients until they are transferred to designated places for EVD care. One of the factors cited to inhibit containment in the 2014–2015 West African outbreak was ignorance or lack of knowledge and preparedness of health professionals. As a result, most health workers misdiagnosed EVD cases as the early symptoms of Ebola resembled those of other diseases such as malaria, cholera and Lassa fever that are endemic in the affected countries [5].

The need to expand the training on EVD beyond clinical nursing staff as observed by the study is of paramount importance. Training both clinical and public health workers will provide a holistic approach to any EVD outbreak. Contact tracing and screening of people at their homes is required in times of outbreak to facilitate containment. This activity in most health systems in developing countries are undertaken by lower level health staff within the public health sector. In Nigeria for example, door-to-door EVD-related education and screening was reported to have played an essential role in the country’s ability to contain the outbreak [44]. Ghana currently operates a Community-based Health Planning and Services strategy where door-to-door health care service is delivered to deprived areas by trained Community Health Officers [45, 46]. This strategy could therefore be harnessed to convey EVD prevention and control messages as well as screening of contacts and members of households for EVD. Focusing on clinical aspects of disease alone is less effective. However, successful integration of prevention and treatment efforts including involving community health workers, who can encourage sick patients to come to health care institutions is reported to be more successful [47].

### Perceptions about the health system preparedness

**Fig. 1:**
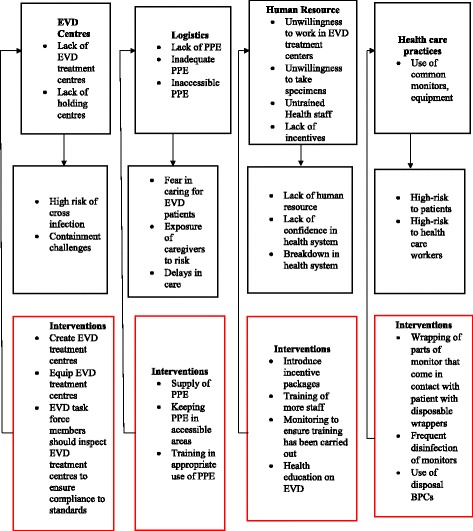
Health system factors, their effect on EVD prevention and containment and ways to address these factors

Four major thematic areas were derived from the data to explain the perceptions about the health system preparedness towards a possible EVD outbreak. These include; provision of Ebola treatment centres, logistics, human resource and health care practices (Fig. 1). The study showed that though efforts are being made to establish EVD treatment centres in various regional and district health facilities, many of such facilities are yet to set up holding centres for suspected EVD cases. Holding places are very important in EVD preparedness and containment [48]. Quick isolation of cases is required to limit the transmission of any infectious condition. In Liberia, Guinea and Sierra Leone, inadequate facilities including treatment centres was reported as a major challenge in containing the outbreak [5]. In Liberia for example, a mathematical model has showed that providing 2400 beds for EVD patients over a period of 2 weeks, while simultaneously increasing case find by five-fold, could have prevented 62, 220 EVD cases by Dec 15, 2014 during the peak of the outbreak in that country [49]. This therefore reiterates the need to have EVD treatment and holding centres across the regions in readiness for any outbreak because of their potential role in the prevention and containment of an outbreak.

Furthermore, a vibrant and well-prepared health system is required to contain health emergencies like an EVD outbreak. Adequate supply of PPE are required for rapid response to EVD cases. In Sierra Leone, limited provision of appropriate PPE and hand washing facilities were reported to be responsible for EVD infections among health care workers [50]. In a review of the experiences of health care providers in previous outbreaks of Ebola Haemorrhagic Fever in Central Africa (1995, 2000 and 2003 outbreaks), the lack of basic protective equipment and other resources to provide health care was reported to have contributed to increasing the risk of infection among health workers [51]. Though respondents generally reported the lack of PPE in their facilities to protect care providers against infection, one practice emerged as problematic in facilities that have been provided with PPEs. This problem is the locking up of PPEs in cupboards whose keys are kept by the “in-charge” of the unit. This practice has the tendency to impede rapid EVD response which is required to curtail transmission of the infection to other people. This practice could also lead to neglect of suspected EVD cases or increased fear and panic in the health facility as found in this study. In a round table discussion among various stakeholders on EVD, it emerged that fear of the condition was influencing the confidence of health workers and the system to handling an outbreak [52]. This practice requires a review to make available PPE to workers at all times in the health facility. It has been observed that the greatest risk of transmission of EVD is not from patients with diagnosed infection but from delayed detection and isolation [11], reinforcing the need for quick response.

### Attitude of health workers to Ebola Virus Disease prevention, case management and containment

The study found that there was an increase in infection prevention and control practices among health workers was reported in this study. This is an important consideration in any EVD prevention and control in the health facility. Frequent hand washing using soap and water or alcohol-based hand sanitizers can readily disrupt the envelope of single-stranded Ribonucleic Acid virus such as Ebola virus [11]. However, the use of common equipment such as cuff of sphygmomanometer and the diaphragm of stethoscope should be of concern to health care practitioners. These items could get contaminated when used on infected persons serving as vehicles for transmission in the health facility. A recent study has showed that the cuff of blood pressure apparatus used for multiple patients carry micro-organisms with bacterial organisms found in 85% of the 120 blood pressure cuffs assessed. The highest rates of contamination (90%) were found in the outpatients department [53]. There is therefore the need for health workers to find innovative ways of reducing direct contact of such equipment to the skin in the event of an outbreak. A disposable wrapper could be used to serve as boundary between the equipment and the skin. Frequent disinfect of such items are also recommended. Though this has been proven to reduce micro-organisms to a greater extent, the use of disposable blood pressure cuff is being strongly recommended since some micro-organisms can survive the disinfection process [54].

Another area of concern which has to be addressed in Ghana’s preparation for EVD is the deep seated perception that health workers who take care of EVD patients stand the risk of being neglected should they become infected. A directive by leaders that health workers who suspect they may be experiencing early signs and symptoms of EVD should stay at home for observation was misconstrued to mean the likelihood of neglecting the health workers who may decide to take of EVD cases. An earlier study has reported that health workers in Ghana held the view that health managers pay less attention to their health issues [55]. There is therefore the need to address this perception as it could accentuate health workers unwillingness to care for Ebola patients.

Majority (86.4%) of the respondents indicated their unwillingness to accept posting to EVD treatment centres should there be an outbreak in Ghana, others (13.6%) could offer to help if incentive are provided. A study among health workers in the United States (US) also found that 25.9% of all participants and 43.6% of nurses were unwilling to provide care to Ebola patients with many expressing concerns about personal risk and danger to friends and family members [56]. This study also found that health workers in Ghana are alarmed about higher risk of infection associated with taking care of Ebola patients and possible transmission of the infection to their families and other associates. Despite the contextual differences between Ghana and the US, similar concerns about risk of infection and unwillingness to care for Ebola patients exist. EVD is a highly fatal condition and many health workers have been infected in both the current and previous outbreaks. For example, in the 1995 Democratic Republic of the Congo outbreak, 250 individuals died from the condition, out of which 47 (approximately 20%) were health care professionals [57]. In the West African outbreak, about 881 health workers were reported to have contracted the infection and more than half of them have died [58]. Insurance package should therefore be designed and incorporated into the current EVD preparation plan [7]. The loss of the lives of many medical staff in the 2014–2015 outbreak is an obstacle to outbreak control measures. This depletes one of the most important assets (human resources) for controlling any outbreak. This has also increased the level of fear, anxiety and sense of hopelessness among the general public [21]. Nonetheless, it is important to educate health workers on the disease as higher knowledge about the condition is believed to be likely to result in positive attitude to Ebola patients [56]. This can also lead to proper use of PPEs thereby minimizing both self and cross infection.

In addition, education and training of health workers on Ebola should emphasize that with high infection prevention and control measures and proper use of PPE, one can take care of EVD cases and remain unaffected. This is because respondents in this study were of the opinion that the use of biohazard suits did not offer full protection against personal risk of infection. This notwithstanding, a contagious parameter predicts a lower EVD transmission with proper use of PPEs than other conditions which health care workers take care of in their facilities. From previous EVD epidemics, it has been determined that primary human case generates between 1 to 3 secondary cases on average [59], as compared to 14–17 for measles outbreaks in West Africa [60]. Therefore, training on appropriate use of PPE and ensuring adequate supply of PPE is important because of its potential role in timely response to outbreak and the decision to accept posting to Ebola treatment centres.

Figure 1 provides a summary of the contextual issues identified in Ghana regarding the health system factors that may adversely affect containment efforts, what role they will play and the interventions to reduce the effects of these factors.

### Limitations of the study

Majority of the participants in this study were nurses. The other health staff that participated in the study were health managers whose work does not require coming into direct contact with patients. Thus it would have been necessary to elicit the perceptions and attitude of other cadre of health workers whose work may require coming into direct contact with patients or their body fluids such as laboratory workers and medical officers. Their perceptions may differ from that of nurses. Nonetheless, the study findings clearly showed the unwillingness of laboratory staff to also attend to Ebola patients in the two classical cases reported in the study.

## Conclusions

The study concludes that preparatory works for EVD prevention and containment in Ghana are perceived as inadequate by health workers. Screening of international travellers is undermined by the use of unapproved entry and exit routes. There was a general perception that majority of health workers have not received training on EVD. Inadequate supply of PPEs and lack of EVD treatment centres was also reported. Therefore, Ghana needs to strengthen preparation in the area of training of health workers, provision and accessibility of PPE and incentives for health workers to better position her to contain and manage EVD cases.

## Abbreviations

AS: Ashanti region
EVD: Ebola Virus Disease
FGD: Focus group discussion
GAR: Greater Accra region
IDI: in-depth interview
NR: Northern region
PPE: Personal Protective Equipment
VR: Volta region
WR: Western region
